# Deep learning-enabled classification of kidney allograft rejection on whole slide histopathologic images

**DOI:** 10.3389/fimmu.2024.1438247

**Published:** 2024-07-05

**Authors:** Yongrong Ye, Liubing Xia, Shicong Yang, You Luo, Zuofu Tang, Yuanqing Li, Lanqing Han, Hanbin Xie, Yong Ren, Ning Na

**Affiliations:** ^1^ Department of Kidney Transplantation, The Third Affiliated Hospital of Sun Yat-sen University, Guangzhou, China; ^2^ Department of Pathology, The First Affiliated Hospital of Sun Yat-sen University, Guangzhou, China; ^3^ School of Automation Science and Engineering, South China University of Technology, Guangzhou, China; ^4^ Research Center for Brain-Computer Interface, Pazhou Lab, Guangzhou, China; ^5^ Center for Artificial Intelligence in Medicine, Research Institute of Tsinghua, Pearl River Delta, Guangzhou, China; ^6^ Department of Anesthesiology, The Third Affiliated Hospital of Sun Yat-sen University, Guangzhou, China; ^7^ Scientific Research Project Department, Guangdong Artificial Intelligence and Digital Economy Laboratory (Guangzhou), Pazhou Lab, Guangzhou, China; ^8^ Shensi lab, Shenzhen Institute for Advanced Study, University of Electronic Science and Technology of China (UESTC), Shenzhen, China; ^9^ The Seventh Affiliated Hospital of Sun Yat-Sen University, Shenzhen, China

**Keywords:** kidney transplantation, artificial intelligence, renal rejection, hematoxylin eosin-stained slides, pathological assessment

## Abstract

**Background:**

Diagnosis of kidney transplant rejection currently relies on manual histopathological assessment, which is subjective and susceptible to inter-observer variability, leading to limited reproducibility. We aim to develop a deep learning system for automated assessment of whole-slide images (WSIs) from kidney allograft biopsies to enable detection and subtyping of rejection and to predict the prognosis of rejection.

**Method:**

We collected H&E-stained WSIs of kidney allograft biopsies at 400x magnification from January 2015 to September 2023 at two hospitals. These biopsy specimens were classified as T cell-mediated rejection, antibody-mediated rejection, and other lesions based on the consensus reached by two experienced transplant pathologists. To achieve feature extraction, feature aggregation, and global classification, we employed multi-instance learning and common convolution neural networks (CNNs). The performance of the developed models was evaluated using various metrics, including confusion matrix, receiver operating characteristic curves, the area under the curve (AUC), classification map, heat map, and pathologist-machine confrontations.

**Results:**

In total, 906 WSIs from 302 kidney allograft biopsies were included for analysis. The model based on multi-instance learning enables detection and subtyping of rejection, named renal rejection artificial intelligence model (RRAIM), with the overall 3-category AUC of 0.798 in the independent test set, which is superior to that of three transplant pathologists under nearly routine assessment conditions. Moreover, the prognosis models accurately predicted graft loss within 1 year following rejection and treatment response for rejection, achieving AUC of 0.936 and 0.756, respectively.

**Conclusion:**

We first developed deep-learning models utilizing multi-instance learning for the detection and subtyping of rejection and prediction of rejection prognosis in kidney allograft biopsies. These models performed well and may be useful in assisting the pathological diagnosis.

## Introduction

Chronic kidney disease is a progressive, irreversible, and incurable disease, with a high prevalence rate and mortality (1). For patients who progress to end-stage renal disease, kidney transplantation (KT) is the only effective treatment (2). Renal allograft transplantation induces immune system activation and may cause rejection. Despite the use of potent immunosuppressants, kidney transplant rejection still poses a significant risk and greatly impacts patient outcomes (3). Moreover, it is considered the primary independent risk factor for long-term allograft survival (4).

The correct diagnosis is crucial in managing rejection, with histopathological assessment of allograft biopsy being the gold standard for diagnosis (5). This assessment usually includes detection, subtyping, and grading of rejection according to Banff criteria (6). The identification of rejection subtypes is significant in diagnosing rejection. Different rejection subtypes are managed quite differently and have varying prognoses (7). However, accurately differentiating between different rejection subtypes can be challenging. In addition, there are some limitations to the pathological assessment.

The current assessment of rejection is a time-consuming, labor-intensive, and expensive process. This process not only involves empirical observation of morphology but also requires immunohistochemical staining (8). Moreover, manual assessment is subjective and susceptible to inter-observer variability, resulting in limited reproducibility (9). Therefore, there are great application prospects for an automated tool to assist in pathological assessment, reducing workload, eliminating bias, and speeding up diagnosis.

Deep learning, which utilizes multiple layers of abstraction to process data, has shown significant advancements in image recognition and is widely applied in medical image analysis and diagnosis (10–12). Compared with traditional machine learning, deep learning has the following advantages: automatic feature learning, processing of large amounts of complex data (including structured and unstructured data), fault tolerance, excellent predictive performance, scalability, and generalization capabilities (10). Numerous studies have demonstrated the diagnostic capabilities of artificial intelligence (AI) models constructed through deep learning in fields like radiology and oncology (13–21). However, the application of deep learning in kidney transplant pathology is limited, with only one previous study focusing on the classification of renal allograft pathology without rejection subtyping (22). This study lacks a connection between pathological characteristics and clinical prognosis, limiting its clinical significance.

Here, we aimed to develop a deep learning system (DLS) for the pathological assessment of kidney transplant rejection. The system enables the detection and subtyping of rejections by scanning only hematoxylin and eosin (H&E) stained whole-slide images (WSIs). Additionally, the DLS visually presents the proportion of rejection subtypes and highlights the regions with distinguishing characteristics in different cases. We also compare the performance of the DLS with pathologists of varying expertise levels in assessing an independent testing dataset. Furthermore, we develop models to predict graft loss and treatment response in patients with rejection for risk stratification.

## Materials and methods

### Ethics approval and participants

The study received approval from the ethics committees of the Third Affiliated Hospital of Sun Yat-sen University (IRB No. [2023]02–041-01). The ethics committees waived the need for written informed consent from participants.

**Figure 1:**
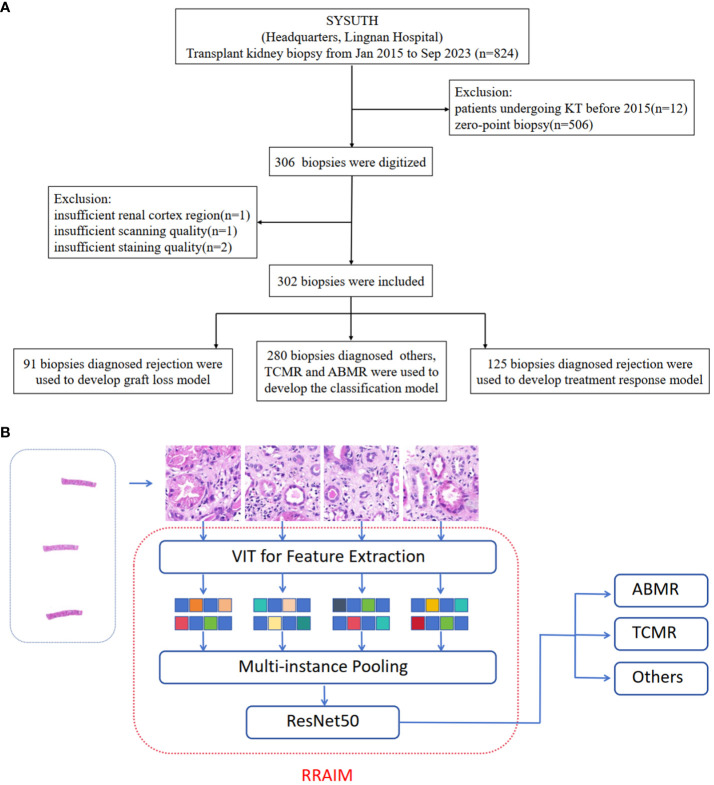
Study design, data curation, and RRAIM classification flowchart. **(A)** Transplant kidney biopsy specimens from two independent hospitals (Headquarters and Lingnan of SYSUTH) were included in this study. After exclusion, a total of 906 WSIs from 302 digital kidney transplant biopsies were used in the analysis. **(B)** Multi-instance learning framework can learn feature extraction, feature aggregation, and global classification at the same time.

The study retrospectively reviewed patients who underwent transplant kidney biopsy at the Headquarters and Lingnan Hospital of the Third Affiliated Hospital of Sun Yat-sen University (SYSUTH) between January 2015 and September 2023. Patients who underwent kidney transplantation before 2015 were excluded and zero-point biopsy and repeat biopsy specimens were excluded. The screening process is shown in **Figure 1A**.

### Data collection and data set curation

Clinical data were obtained from the hospital information system, while pathological data were acquired from written pathology reports. Patients with loss of graft function were considered graft loss (23). Patients were considered respondence for rejection treatment if the estimated glomerular filtration rate (eGFR) returned to within 10% of baseline level 3 months after biopsy (24). The baseline level of eGFR is determined by assessing eGFR within 3 months before the onset of rejection. The eGFR of patients was calculated using the Chronic Kidney Disease-Epidemiology (CKD-EPI) algorithm (25).

A total of 906 WSIs were obtained from 302 allograft biopsy specimens. These specimens were classified based on the consensus of two experienced transplant pathologists according to Banff 2019 criteria, which are presented in **Supplementary Table 1** (8). Out of the total, 125 cases were classified as rejection (Banff category 2–4), which included TCMR, ABMR, and mixed TCMR/ABMR. The remaining 177 cases were categorized as other lesions (Banff category 1 and 5). Cases that were borderline TCMR, and suspicious ABMR were also classified as rejection categories.

H&E-stained specimen slides were scanned at a magnification of x40 using a digital slide scanning instrument (Panoramic 250 FLASH, 3DHISTECH Ltd, Budapest, Hungary). Specimens with quality issues such as insufficient renal cortex region, repeated scanning with failed focalization, or faded staining were excluded. Digital sections were utilized in the development of the model. Included cases were randomly assigned to training sets and testing sets, with proportions of 70% and 30%, respectively. A total of 280 biopsies diagnosed with other lesions, TCMR, and ABMR were used to develop the classification model. The graft loss model and treatment response model were developed using 91 and 125 cases diagnosed with rejection respectively. The graft loss model is used to predict graft loss within 1 year after rejection, and its dataset does not include 34 cases with less than 1 year of follow-up after rejection. The composition of the training set and testing set in different models is shown in **Supplementary Table 2**.

### Development of deep learning models by multi-instance learning

We annotated typical tissue as the region of interest (ROI) and outlined it precisely with the automated slide analysis platform (ASAP 1.9) software, which was reviewed by an expert pathologist. The position coordinates were saved in the XML format, and a corresponding mask was generated with the same pyramid resolution as the whole-slide image (WSI).

Digital pathological WSI can be up to megapixels in size, which can easily lead to insufficient memory as input. Therefore, we first divide the WSI into image blocks of appropriate size to meet the needs of subsequent operations. Considering the high level of detail of the pathological image, we choose a relatively small size of 515 * 512 and control the spacing to avoid overlap, to ensure that the segmented image block can represent the whole region of the whole slice. We use the Openslide image pyramid and sliding window function to extract image blocks. Firstly, the pathological WSI is loaded, and the pyramid level is set to 400X. Secondly, the sliding window size is set to 515* 512, and there is no overlapping sampling. Finally, the image of each position in the scanning process is saved as Patch. Repeat the above process, and finally get all the patches of the WSI. Considering the non-overlap between image blocks, the problem of repetitive feature learning is avoided, which helps improve the efficiency of multi-instance learning.

After obtaining the patches, we use the visual transformer network (Vision Transformer, ViT) to extract the features of each patch. The main reason is that ViT has a good ability to learn local and global information of the image, and can obtain high-quality feature expression. Specifically, we load the ViT model pre-trained on ImageNet, because of its large number of parameters, it is difficult to converge if we train the transformer from scratch. To speed up the model training, we fix the pre-training parameters and only use ViT as a feature extractor. For each image block, the average pool feature extracted by ViT is a 768-dim vector. Repeat this process, and finally output a n * 768 feature matrix, representing all the features of a WSI. Where n is the total number of patches for the WSI.

After obtaining the features of each patch of the WSI, we construct the framework of a two-layer learner. The first layer is a designed multi-instance pooling layer, and the second layer is a ResNet50-based classifier. The function of the multi-instance pooling layer is to aggregate the characteristic information of all patches in a WSI. Here we use average pooling, that is, averaging the eigenmatrix. After pooling, a 768-dim vector corresponding to a WSI is obtained. The WSI-level vector splices the WSI features and inputs them into the second-layer classifier. We construct ResNet50, remove its original fully connected sublayer, and finally add a softmax fully connected sublayer containing two neurons or three neurons to output the prediction probability of two categories or three categories. We first carry out pre-training on the ImageNet data set. Then use the WSI feature to fine-tune the last sublayer to complete the final two-classification or three-classification training. Such a framework can learn feature extraction, feature aggregation, and global classification at the same time, and truly realize end-to-end multi-instance learning. The whole process is illustrated in **Figure 1B**.

### Visualization of panoramic WSI

The ensemble model yielded patch-level probabilities, and the specific coordinates of these patches within the patient’s WSI were acquired through the partitioning procedure and XML file. Utilizing the matplotlib library (https://matplotlib.org/), Each patch was represented by a color, where yellow, red, and black represent that the patch belongs to other lesions, ABMR, and TCMR, respectively. By reassembling the patches based on their coordinates, a panoramic picture of the patient’s response density toward the WSI was generated, providing a visual representation. The methodology of three CNN-based models (InceptionV3, ResNet50, and EfficientNet-B5) and the ensemble model are provided in the **Supplementary Material**.

### Biopsy specimen reviews by 3 pathologists for comparison

Comparisons of DLS’s decisions of three-classifications with those of three pathologists were carried out on an internal testing set. These pathologists all received transplant pathology training and were divided into junior, intermediate, and senior levels according to different clinical experiences (1–2 years, 4–5 years, and more than 10 years). In addition, they were not involved in the selection and labeling of the cases and independently assessed each case from the testing set.

In the first assessment, the pathologists only considered H&E-stained WSIs to make their decision. During the second assessment, they also assessed the corresponding Periodic Acid-Schiff (PAS), Periodic-acid silver methenamine (PASM), Masson stained WSI, and the results of immunofluorescence, immunohistochemistry, and electron microscopy to make their decision. The referral decisions made by the DLS were then compared with the decisions made by the pathologists during both assessments.

### Statistical analysis

To evaluate the classification performance of the patch-based models on the test dataset, we examined the receiver operating characteristic (ROC) curve. This curve was constructed by plotting the true positive rate (TPR, sensitivity) against the false positive rate (FPR, 1-specificity) across different threshold values. The accuracy (ACC) of the models was quantified by calculating the area under the ROC curve (AUC). Additionally, performance measures such as ACC, sensitivity (SENS), specificity (SPEC), positive predictive value (PPV, precision), and negative predictive value (NPV) were computed using confusion matrices. The F1 score, which represents the harmonic mean of precision and sensitivity, was employed to assess model performance.

Clinicopathological data of patients were expressed as frequencies for categorical variables and medians [interquartile range] for continuous variables. Statistical analysis was performed using SPSS version 25.0.

## Results

### Population characteristic

A total of 302 patients who had undergone post-transplantation biopsy and had corresponding available WSI were included in the analysis. The characteristics and pathological diagnoses of all patients are presented in **Table 1** and **Supplementary Table 3**, respectively. Among the population, 41.4% (125/302) of patients with rejection, while 58.6% (177/302) of patients without rejection. The median months from transplantation to biopsy in other lesions, TCMR, ABMR, and mixed rejection were 15.3(4.5, 41.8), 6.4(3.6, 12.7), and 36(5.9, 62.8), and 19.3(6.1,47.5), respectively. The median months from transplantation to biopsy in other lesions, TCMR, ABMR, and mixed rejection were 15.3(4.5, 41.8), 6.4(3.6, 12.7), 36(5.9, 62.8), and 19.3(6.1,47.5), respectively.

**Table 1:** Distribution of the patient characteristics.

Characteristics	Other lesions (N=177)	Rejection(N=125)		
		TCMR (N=57)	ABMR (N=46)	Mixed rejections (N=22)
Male sex	130(73)	37(65)	31(67)	14(64)
Age	38(32,47)	36(31,44.5)	44.5(35.8,56)	41.5(37,55.5)
Months from transplantation to biopsy	15.3(4.5,41.8)	6.3(3.6,12.7)	36(5.9,62.8)	19.3(6.1,47.5)
Months of follow-up after biopsy	21.8(3.4,51.9)	17.8(3.2,49.4)	4.6(3.2,14.7)	3(0,5.6)
Graft loss	68(38)	25(44)	12(26)	11(50)
Treatment response		28(49)	25(54)	4(18)
DSA				
Absent	177(100)	54(95)	28(61)	9(41)
HLA I class	0	0	1(2)	0(0)
HLA II class	0	0	6(13)	4(18)
HLA I&II class	0	0	2(4)	1(5)
No available	0	3(5)	9(20)	8(36)
C4d score				
0	163(92)	57(100)	15(33)	12(54)
1	13(7)	0	16(35)	3(14)
2	1(1)	0	9(19)	2(9)
3	0	0	6(13)	5(23)

TCMR, T cell-mediated rejection; ABMR, antibody-mediated rejection; DSA, donor-specific antibody.

### Performance evaluation of classification model based on multi-instance learning

**Figure 2:**
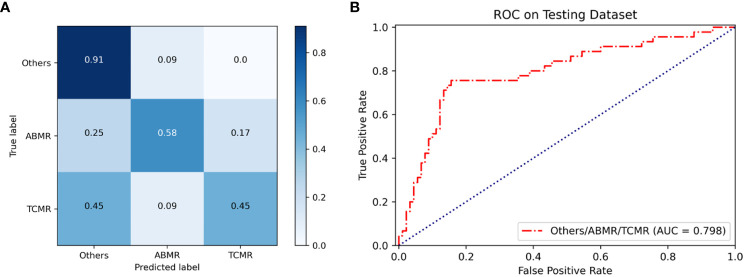
Performance of the classification model based on multi-instance learning. **(A)** The confusion matrix of the model shows the classification results on the internal testing set, from which the overall ACC can be calculated to be 0.71. Numbers represent the percentage of classified correctly (diagonal) and incorrectly (off the diagonal). Blue shading has a positive correlation with classification value and diagnostic accuracy. **(B)** The ROC curve and AUC value of the model (red line) used for three classifications on the internal testing set, the AUC value is 0.798.

Based on an internal testing set, the confusion matrices of the classification model and the three classification results (other lesions, TCMR, and ABMR) of kidney allograft biopsies are shown in **Figure 2**. Diagonal elements represent the percentage for which the model predictions are consistent with the actual diagnosis, while off-diagonal elements represent the percentage for which the two are inconsistent. In the testing set, the overall AUC and ACC values of the model are as follows: AUC=0.798, ACC=0.71. The classification model was named the renal rejection artificial intelligence model (RRAIM), and its performance parameters are presented in **Table 2**.

**Table 2:** Performance parameters of the classification model based on multi-instance learning.

Model	Classification	Precision	SENS	F1 scores	Overall ACC
Classification model	Others	0.71	0.91	0.80	0.71
	ABMR	0.70	0.58	0.64	
	TCMR	0.71	0.45	0.56	

TCMR, T cell-mediated rejection; ABMR, antibody-mediated rejection.

### Performance evaluation of three CNN-based classification models and the ensemble model

Three CNN-based models (InceptionV3, ResNet50, and EfficientNet-B5) and the ensemble model also were trained to differentiate the three classifications of kidney allograft biopsy. The confusion matrices and ROC curves of these models at the patch level are shown in **Supplementary Figure 1**. The overall AUC of three basic models (InceptionV3, ResNet50, and EfficientNet-B5) and the ensemble model predicting three categories at the patch level are respectively 0.765, 0.789, 0.775, and 0.799. As seen in **Supplementary Figure 2**, the overall AUC and ACC of the ensemble model predicting three categories at the patient level are respectively 0.863 and 0.70. The performance parameters of these models are presented in **Supplementary Table 4**.

### Classification map of biopsy specimen in whole-slide image level

**Figure 3:**
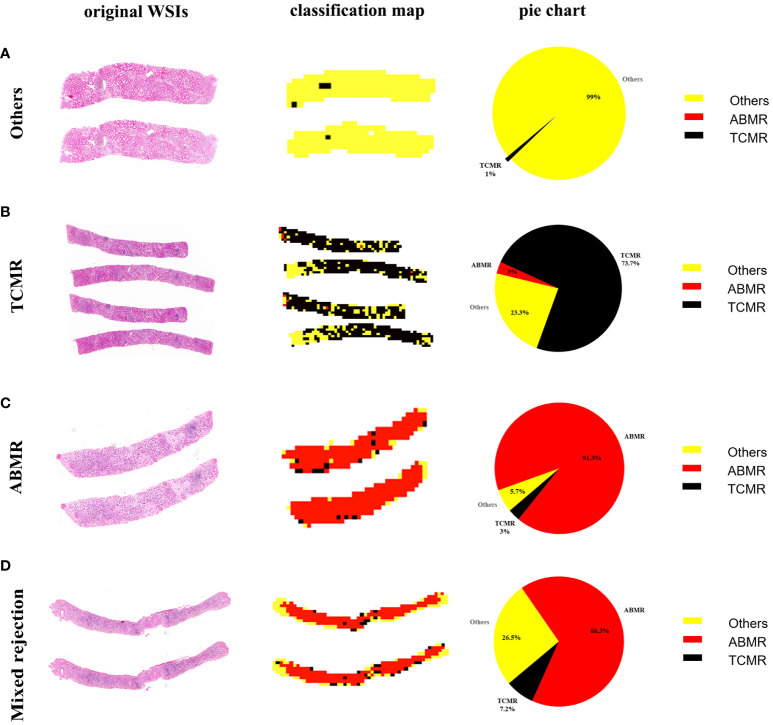
WSI-level panoramic classification map of biopsied specimens: **(A)** Others, **(B)** TCMR, **(C)** ABMR, **(D)** mixed rejection. (Left) Original whole-slide images. (Middle) The classification maps’ different colors represent different lesions: the yellow area represents other lesions, the black area represents TCMR, and the red area represents ABMR. (Right) The pie charts quantitatively show the percentages of different categories within each WSI.

Panoramic classification maps were generated using representative WSIs for each category in biopsy specimens. Here, areas of different categories throughout the WSI from the H&E-stained section can be exhibited by using different colors, even in mixed rejection cases where TCMR coexists with ABMR. The proportion of various categories is visually displayed in a pie chart to achieve a quantitative analysis of WSI. As seen in **Figure 3**, there is a small proportion of misclassifications, such as TCMR appearing in the classification map of other lesions (1% black region), ABMR appearing in the classification map of TCMR (3% red region), and TCMR appearing in the classification map of ABMR (3% black region). These misclassifications are likely due to interference from sclerotic glomeruli, atrophic tubules, and proliferated fibrous tissue.

### Heat map of biopsy specimen in patch level

**Figure 4:**
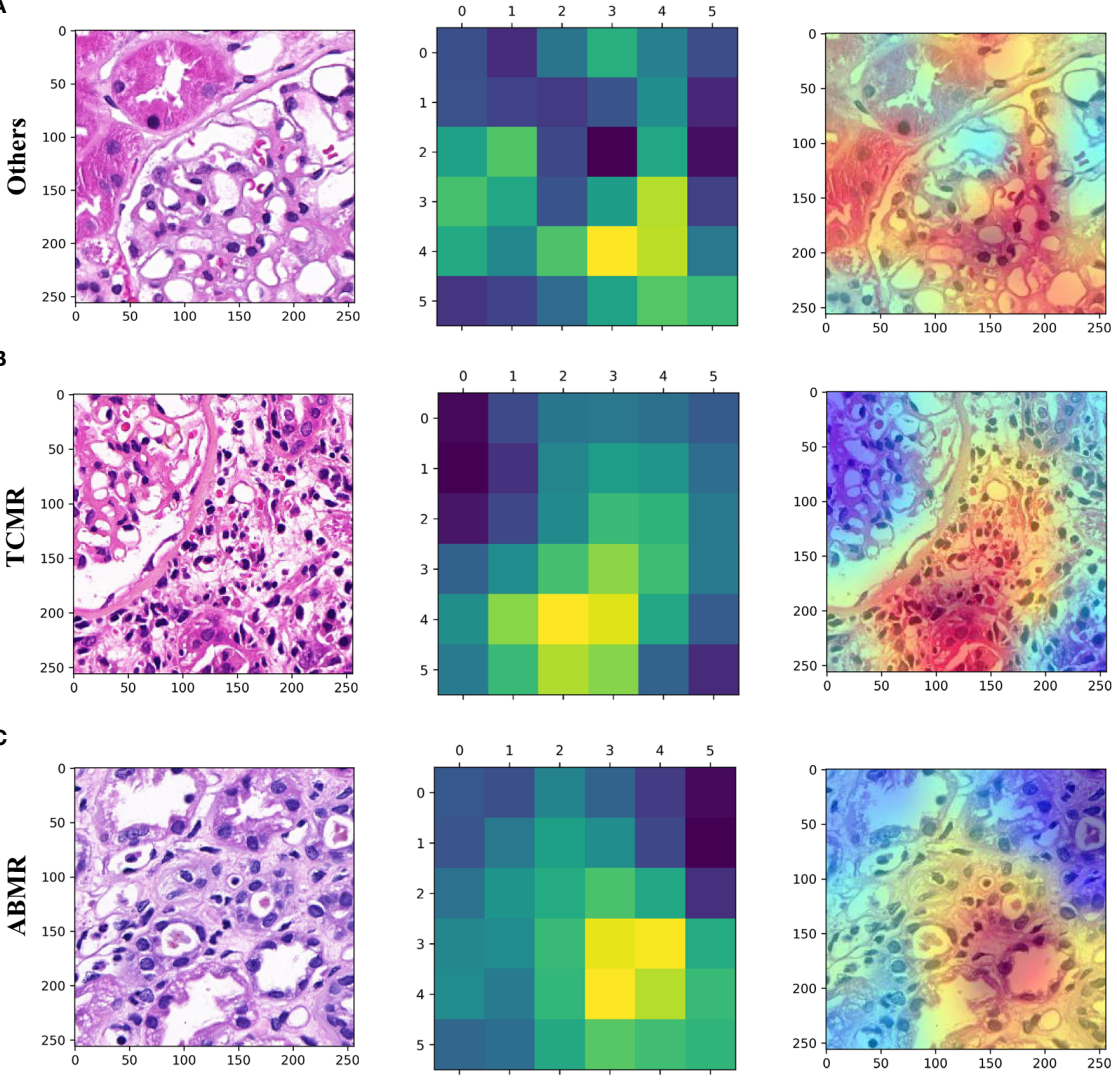
Patch-level hot maps. **(A)** (Others), **(B)** (TCMR), **(C)** (ABMR). (left) Original whole-slide images. (middle and right) GradCAM visualizes the learning process and extracts discriminative features of interest (highlighted areas).

Utilizing the trained Convolutional Neural Network (CNN), the visual gradient weighted class activation mapping (Grad-CAM) technique was employed to generate heat maps to discriminate three types of lesions. As shown in the heat map (**Figure 4**), interstitial inflammation and tubulitis in WSI from the TCMR case and partial peritubular capillaritis in WSI from the ABMR case are highlighted.

### Comparison between deep learning and pathologists

**Figure 5:**
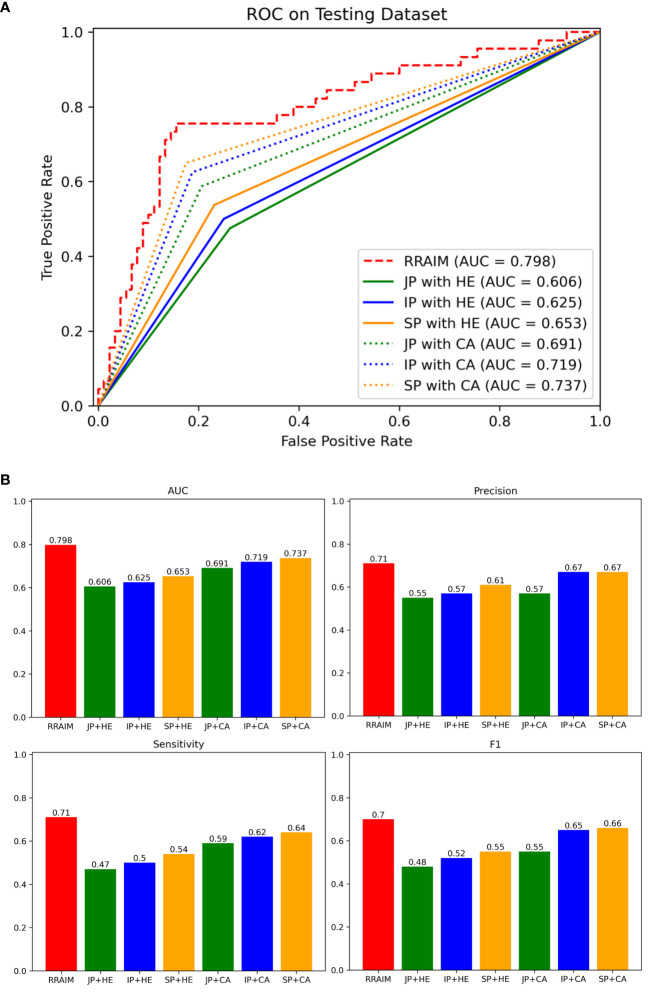
Comparisons of diagnostic performance of RRAIM with pathologists. **(A)** The ROC curves and AUC values of pathologists of all levels equipped with different diagnostic conditions on the internal testing set: junior pathologist assessed with H&E stained WSIs (JP with HE, green line), intermediate pathologist assessed with H&E stained WSIs (IP with HE, blue line), senior pathologist assessed with H&E stained WSIs (SP with HE, orange line), junior pathologist with comprehensive assessment (JP with CA, green dotted line), intermediate pathologist with comprehensive assessment (IP with CA, blue dotted line), and senior pathologist with comprehensive assessment (SP with CA, orange dotted line). **(B)** The AUC values (0.798, 0.606, 0.625, 0.653, 0.691, 0.719, and 0.737), precision (0.71, 0.55, 0.57, 0.61, 0.57, 0.67, and 0.67), sensitivity (0.71, 0.47, 0.5, 0.54, 0.59, 0.62, and 0.64), and F1 scores (0.7, 0.48, 0.52, 0.55, 0.55, 0.65, and 0.66) of RRAIM and pathologists of all levels equipped with different diagnostic conditions (JP with HE, IP with HE, SP with HE, JP with CA, IP with CA, and SP with CA) are displayed in histograms.

We used an internal testing dataset to evaluate the diagnostic performance of RRAIM and compared it with that of three pathologists under different conditions. As shown in **Figure 5**, RRAIM outperforms pathologists at all levels with different diagnostic conditions in terms of AUC, precision, sensitivity, and F1 score for predicting three classifications of kidney transplant biopsy. In the case of three pathologists assessing H&E-stained WSI, a junior, intermediate, and senior subspecialty pathologist achieved AUC of 0.606, 0.625, and 0.653, respectively. When simultaneously assessing PAS, PASM, and Masson stained WSIs, as well as immunohistochemistry and electron microscopy results, a junior, intermediate, and senior pathologist achieved AUC of 0.691, 0.719, and 0.737, respectively.

### Prediction graft prognosis in rejection cohort

**Figure 6:**
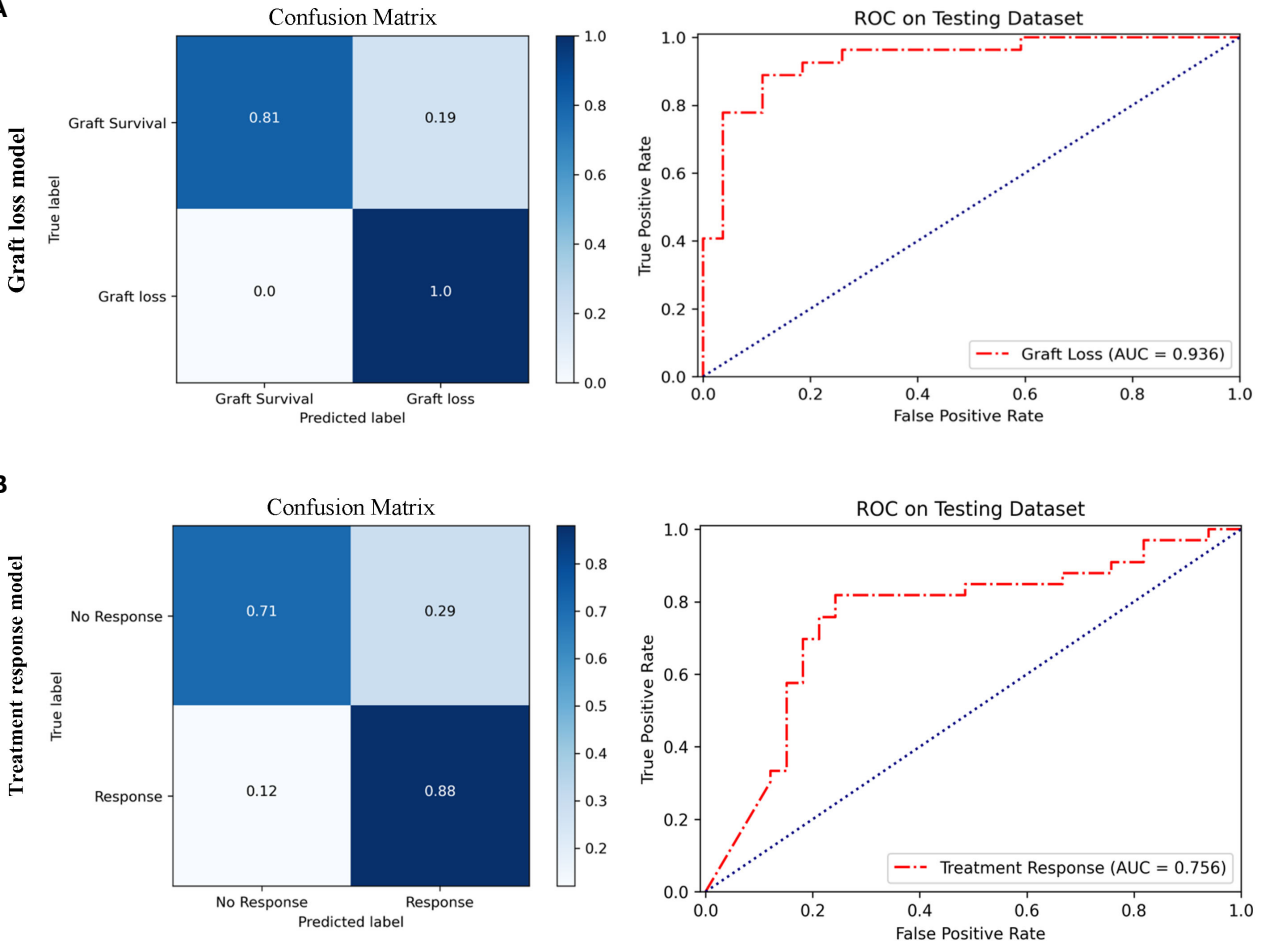
Performance of rejection prognosis models based on multi-instance learning. **(A)** Confusion matrix and ROC curve of the graft loss model for predicting graft loss within 1 year after rejection, the AUC value is 0.936. **(B)** Confusion matrix and ROC curve of the treatment response model for predicting treatment response in rejection, the AUC value is 0.756.

Based on multi-instance learning, two separate models were trained to predict graft loss within 1 year following rejection and treatment response for rejection. The baseline information of patients for the two models is presented in **Supplementary Tables 5**, **6** respectively. As shown in **Figure 6A**, the performance of the graft loss model was assessed based on its AUC and ACC, with values of 0.936 and 0.89, respectively. As shown in **Figure 6B**, the AUC and ACC of the treatment response model are respectively 0.756 and 0.79. The performance parameters of rejection prognosis models based on multi-instance learning are presented in **Table 3**. The ensemble method of three CNNs was also used to train rejection prognosis models, the performance of rejection prognosis models based on the ensemble of three CNNs at the patient level is shown in **Supplementary Figure 3**.

**Table 3:** Performance parameters of the rejection prognosis models based on multi-instance learning.

Models	ACC	Precision	NPV	SENS	SPEC	F1 scores
Graft loss	0.89	0.79	1.0	1.0	0.81	0.89
Treatment response	0.79	0.74	0.86	0.88	0.71	0.79

## Discussion

Transplant pathology plays a crucial role in diagnosing kidney allograft rejection. However, the current diagnostic criteria for rejection, known as the Banff criteria, have become increasingly complex over the past 30 years, making the entire pathological diagnosis process more time-consuming, expensive, and labor-intensive. To address these challenges, the combination of digital pathology and AI offers a potential solution. Here we present a supervised DLS for the classification of kidney allograft biopsy using H&E-stained WSIs, designated as RRAIM. Moreover, RRAIM is the first DLS for the detection and subtyping of kidney allograft rejection.

The current assessment of kidney allograft biopsy is complex and involves assessing multiple staining, including H&E, PAS, PASM, and Masson (8). This is related to the fact that different pathological features are more easily distinguished by the naked eye under different staining. Compared with naked-eye observation, AI can detect hidden morphology or internal features (26), and obtain more information for distinguishing types of kidney allograft lesions even with a single type of staining. Here, we used only H&E-stained WSI to train deep learning models due to H&E staining being the most common stain in kidney allograft pathology and providing clear morphological and structural characteristics. Moreover, RRAIM completes the diagnosis task in less than 30 seconds per case, and processing and H&E staining per biopsy only take one day. Therefore, RRAIM enables rapid and convenient screening of rejection in kidney allograft biopsies.

The RRAIM enables differentiation of TCMR, ABMR, and other lesions, thereby simultaneously enabling the detection and subtyping of rejection. The different rejection subtypes have varying prognoses, so accurate differential diagnosis is crucial. TCMR can be reversed with prompt treatment in most cases, while ABMR often leads to graft loss (4). However, the diagnosis and identification of rejection is challenging, especially in complex or atypical cases, and inexperienced pathologists may easily make incorrect diagnoses. RRAIM uses the latest ViT network for feature extraction, which is particularly suitable for extracting local and global features (27), thereby improving the performance of RRAIM. The patch used by ViT is larger than that of most studies (512x512 pixels at 40x magnification vs. 224x224 pixels), which is closer to reality and may contribute to more reliable results. Moreover, the framework of RRAIM employs multi-instance learning, which is more suitable for obtaining effective distinguishing features from WSI that lacks pixel-level labeling (28). In the rejection prognosis models, the accuracy of the models using multi-instance learning was significantly better than the multi-network ensemble model (0.89 vs 0.63; 0.79 vs 0.62). In the classification model, the overall accuracy of RRAIM is slightly better than the multi-network ensemble model (0.71 vs 0.70). Our results preliminarily confirm the advantages of multi-instance learning architecture for the classification of kidney transplant rejection.

Classification maps and heat maps provide visual outputs for the models. The concurrent appearance of different lesions in WSI can be exhibited through the classification maps, and the quantitative analysis of different lesions in WSI can also be provided by the classification maps. In addition, the patch-level heatmaps highlight areas that are features focused by the model making classification decisions. We found that these features are consistent with the pathological changes of TCMR and ABMR, which provides some interpretability for the deep learning models. The visualization technique may provide transplant pathologists with a useful diagnostic support system, but its value needs further evaluation.

The RRAIM showed better classification performance compared with the three pathologists. When evaluated by three pathologists with only HE-stained WSIs, the performance of RRAIM for classification is superior to them. Furthermore, when evaluated by three pathologists under nearly routine conditions (excluding clinical information), their performance remained inferior to RRAIM. However, only one pathologist of the same level cannot make a statistically significant comparison with RRAIM. Regrettably, recruiting more transplant pathologists for the comparisons is difficult due to the relative scarcity of transplant pathologists.

The rejection prognosis models using multi-instance learning can accurately predict the prognosis of rejection, especially the graft loss within 1 year after rejection. Some rejections lack response to treatment and may lead to short-term graft loss (29, 30). However, these rejections are difficult to identify by clinical indicators alone. We found that pathological features extracted using multi-instance learning have excellent effects on predicting rejection prognosis. Moreover, these models aid in the risk stratification of patients with rejection and guide subsequent treatment and management.

There are some limitations in our study. First, postoperative allograft biopsy samples are limited due to the high bleeding risk of kidney biopsy. In addition, there are difficulties in including more centers due to informed consent and strict data management policies. Second, although our study has confirmed that DLS based on HE-stained WSI enables the detection and subtyping of rejection, the performance of DLS may be affected by the sample size. Larger multicenter studies are required, and we are seeking to partner with more centers. Third, the cases of mixed rejection were relatively few and were not included in the development of the classification model. Further studies with larger sample sizes are required to detect mixed rejection accurately. Fourth, our study did not involve the task of grading rejection by severity, and further follow-up research is needed.

In conclusion, our study demonstrates that it is feasible to use multi-instance learning to detect and subtype renal rejection and to predict rejection prognosis based on H&E-stained WSI. Additionally, DLS-based visualization technology has the potential value to enhance the digital pathology workflow.

## Data availability statement

The original contributions presented in the study are included in the article/**Supplementary Material**. Further inquiries can be directed to the corresponding authors.

## Ethics statement

The studies involving humans were approved by the ethics committees of the Third Affiliated Hospital of Sun Yat-sen University. The studies were conducted in accordance with the local legislation and institutional requirements. Written informed consent for participation was not required from the participants or the participants’ legal guardians/next of kin in accordance with the national legislation and institutional requirements. Written informed consent was not obtained from the individual(s) for the publication of any potentially identifiable images or data included in this article because the ethics committees waived the need for written informed consent from participants.

## Author contributions

YY: Conceptualization, Data curation, Investigation, Methodology, Project administration, Writing – original draft, Writing – review & editing. LX: Data curation, Investigation, Methodology, Writing – original draft. SY: Methodology, Supervision, Writing – original draft. YoL: Data curation, Writing – original draft. ZT: Data curation, Writing – original draft. YuL: Methodology, Writing – original draft. LH: Methodology, Writing – original draft. HX: Conceptualization, Investigation, Writing – review & editing. YR: Formal analysis, Methodology, Visualization, Writing – review & editing. NN: Conceptualization, Investigation, Project administration, Writing – review & editing.
