# Supplementary material for: Deep learning-enabled classification of kidney allograft rejection on whole slide histopathologic images
Source: Front Immunol. 2024 Jul 5;15:1438247. doi: 10.3389/fimmu.2024.1438247 (PMC11257957; doi:10.3389/fimmu.2024.1438247)
Supplement: Supplementary file 1 [file DataSheet_1.docx]

Supplementary Material

# Supplementary Method

**Base classification models and the ensemble model**

Convolutional neural networks (CNNs) are one of the most effective methods for processing image data. Three CNN-based models, including Inception V3, ResNet50, and EfficientNet-B5, were applied to fetch the potential features from WSI patches and to distinguish cruel information for classification. A noteworthy advantage of the EfficientNet-B5 was its trade-off between fewer parameters and a higher ImageNet (<https://image-net.org/>) Top-1 accuracy.

To avoid overfitting, we adopted transfer learning with a relatively small training dataset to train models. Before training, the ImageNet dataset with well-trained parameters was utilized for the first initiation of the three CNN-based models, and then the models were updated during training by backpropagation. A fully connected layer with merely two neurons was used to take the place of the last layers of the three base models. During the training process consisting of 100 epochs, we utilized the Adam optimizer with a learning rate set at 8e-4. The loss function employed was binary cross-entropy, while the activation function used was sigmoid. The input patch size was defined as 256 × 256. The resulting output comprised three classes: the first class represented the likelihood of Others, the second class indicated the likelihood of ABMR, and the third class indicated the likelihood of TCMR for each patch.

The predicted score for each patient was calculated by averaging the predicted probabilities of all patches. By averaging the three scores of the three models for patients, an ensemble model was also created and utilized to predict the patient's final score. Based on the testing set, the predictive performance of three basic models and an ensemble model was compared.

# Supplementary Figures and Tables

## Supplementary Figures


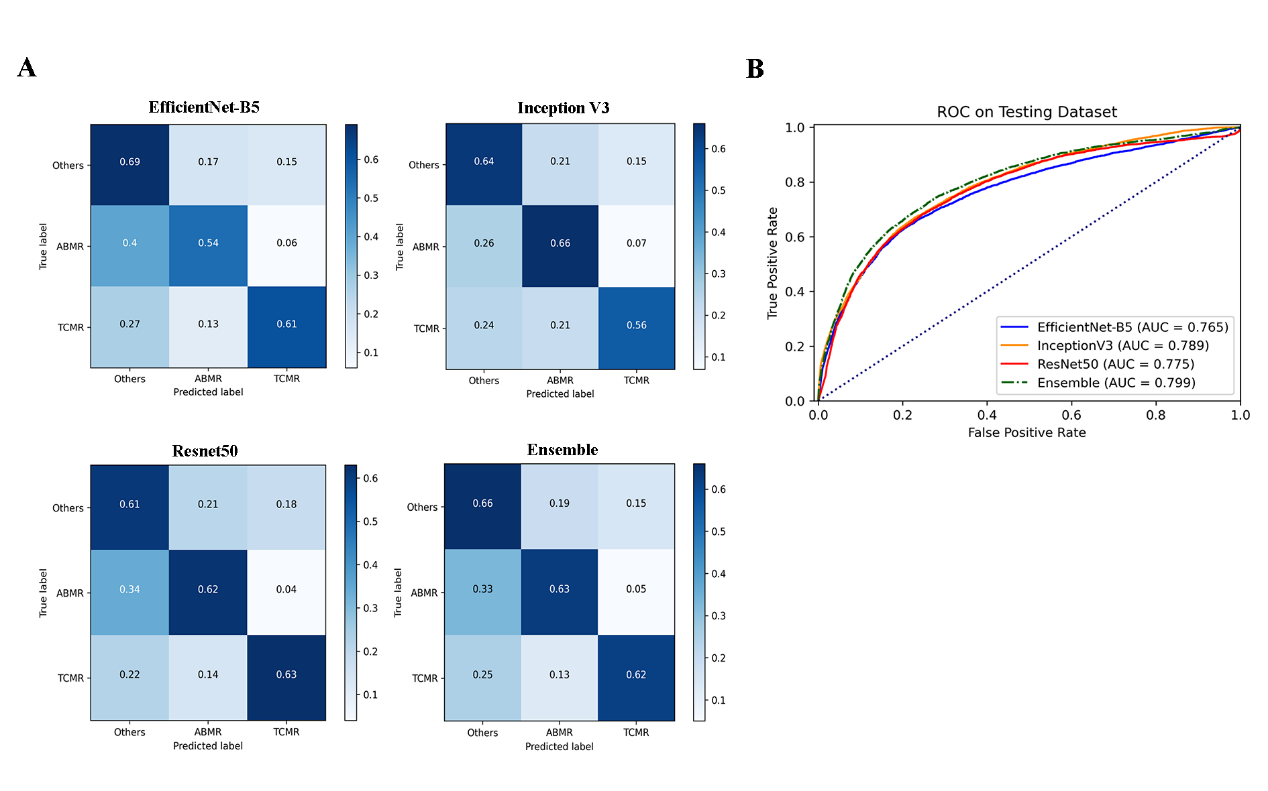


Supplement Figure 1. Performance of deep learning models at the patch level. (A) The confusion matrices for EfficientNet-B5, Inception V3, ResNet50, and Ensemble models show the classification results on the internal testing set. (B) The ROC curves and AUC values for models of EfficientNet-B5 (blue line), Inception V3(orange line), ResNet50 (red line), and Ensemble (green line) on the internal testing set, the AUC values are 0.765, 0.789, 0.775, and 0.799, respectively.


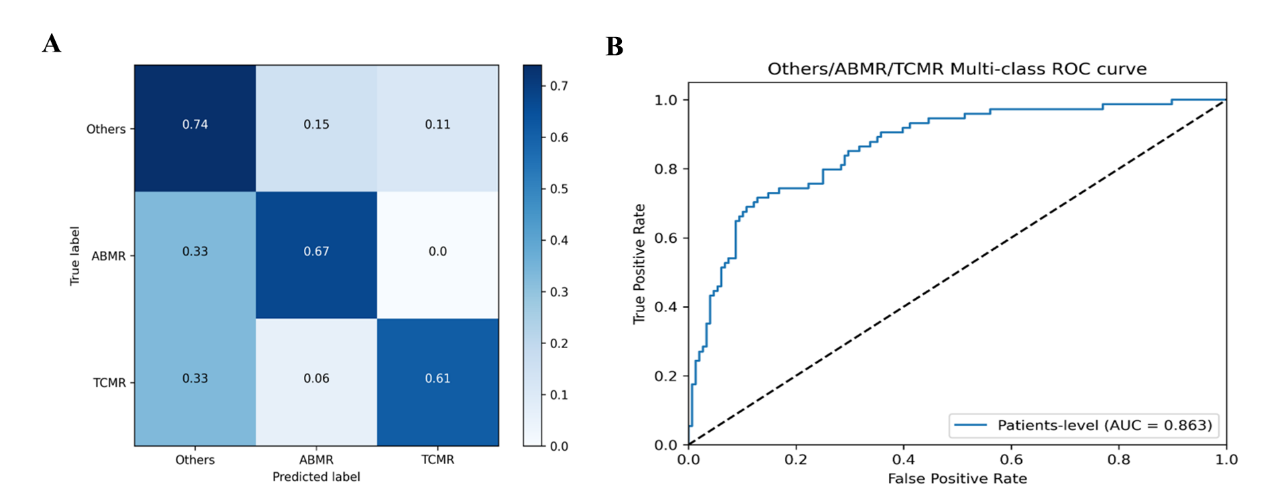


Supplement Figure 2. Performance of deep learning model at the patient level. (A) The confusion matrix of the Ensemble model shows the classification results on the internal testing set. (B) The ROC curve and AUC value of the model (blue line) used for three classifications on the internal testing set, the AUC value is 0.863.


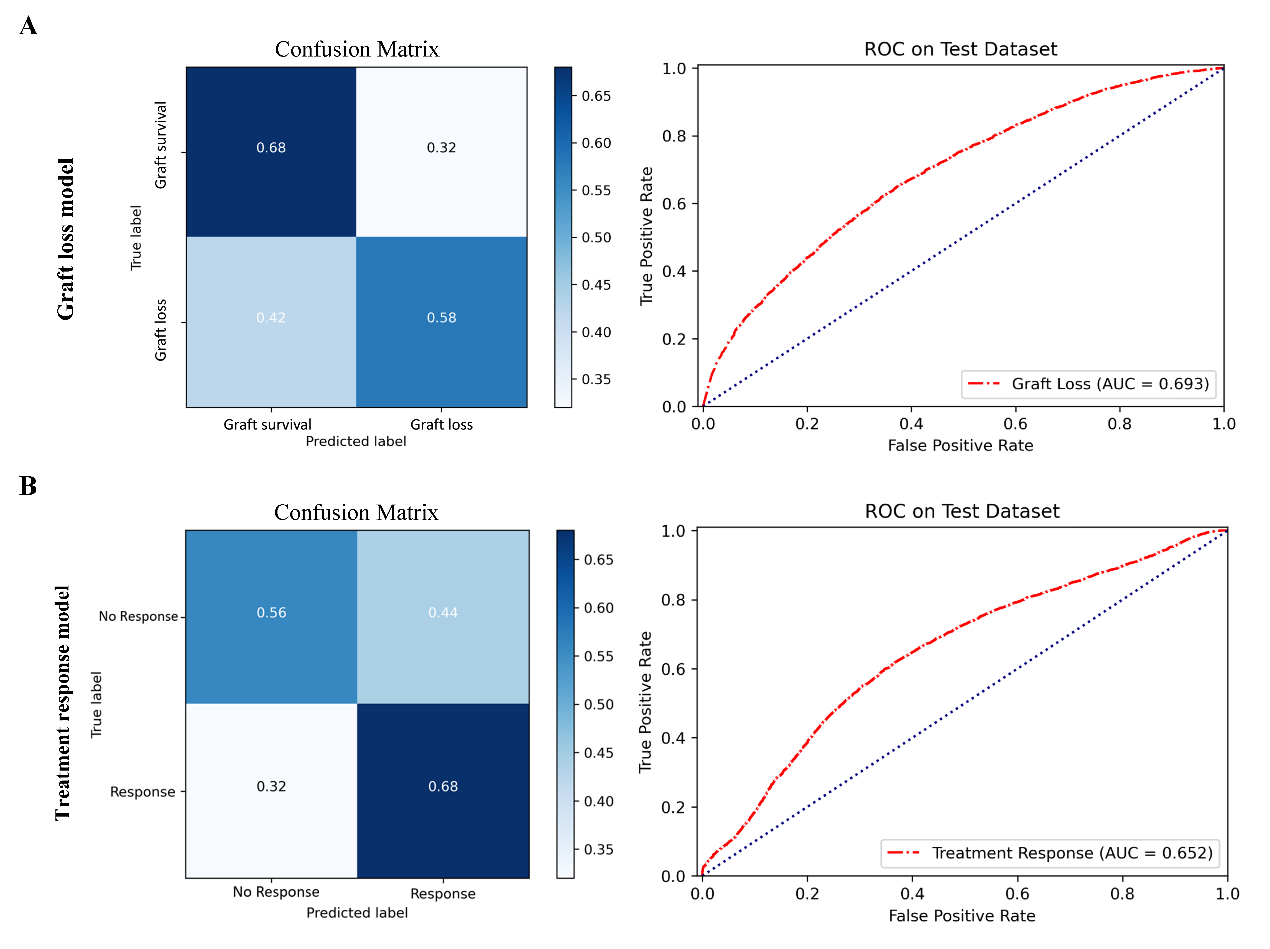


Supplement Figure 3. Performance of rejection prognosis models based on the ensemble of three CNNs at the patient level. (A) Confusion matrix and ROC curve of the graft loss model for predicting graft loss within 1 year after rejection, the AUC value is 0.693. (B) Confusion matrix and ROC curve of the treatment response model for predicting treatment response in rejection, the AUC value is 0.652.

## Supplementary Tables

Supplement Table 1. The classification categories and their diagnostic descriptions of Banff 2019 as used in this study.

| Category | Diagnostic descriptions |
| --- | --- |
| 1 normal biopsy or nonspecific change | This category includes only nonspecific changes. |
| 2 antibody-mediated changes (ABMR) | This category includes active ABMR and chronic active ABMR, with characteristics of microvascular inflammation and/or detective donor-specific antibodies and/or positive C4d staining in peritubular capillaries. |
| 3 borderline T cell-mediated rejection (TCMR) | This category includes cases with scores of tubulitis (t) ≥1 and scores of interstitial inflammation (i)=1 or t=1 and i≥2. |
| 4 TCMR | This category includes Grade I, Grade II, and Grade III of acute TCMR and Grade I and Grade II of chronic active TCMR. |
| 5 Polyomavirus nephropathy | This category includes cases with positive SV40 staining and/or scores of chronic interstitial fibrosis (ci)≥1. |

Supplement Table 2. The composition of the training set and testing set in different models.

|  | Classification model | | |  | Graft loss model | |  | Treatment response model | |
| --- | --- | --- | --- | --- | --- | --- | --- | --- | --- |
|  | Other | TCMR | ABMR |  | Graft loss | No graft loss |  | Response | No response |
| Training set | 124 | 40 | 32 |  | 26 | 38 |  | 40 | 48 |
| Testing set | 53 | 17 | 14 |  | 11 | 16 |  | 17 | 20 |

Supplement Table 3. The pathological diagnosis of the patients.

| category | Diagnosis | cases |
| --- | --- | --- |
| Other lesions (n=177) | Chronic and non-specific change | 64 |
|  | Recurrent IgA nephropathy | 35 |
|  | Polyomavirus nephropathy | 21 |
|  | acute tubular injury | 20 |
|  | Recurrent focal segmental glomerulosclerosis | 12 |
|  | Hypertension-associated injury | 6 |
|  | Diabetic nephropathy | 5 |
|  | Calcineurin inhibitor-associated injury | 4 |
|  | Other recurrent nephropathy | 3 |
|  | Oxalate nephropathy | 2 |
|  | Acute interstitial nephritis | 2 |
|  | Alport syndrome kidney injury | 1 |
|  | Thrombotic microangiopathy | 1 |
|  | Renal cortical necrosis | 1 |
| Rejection (n=125) | ABMR | 46 |
|  | Borderline TCMR | 29 |
|  | TCMR grade I/ II/III | 28 |
|  | Mixed TCMR/ABMR | 22 |

Supplement Table 4. Performance parameters of three basic models and the ensemble model.

| Models | Classification | Precision | SENS | F1 scores | Overall ACC |
| --- | --- | --- | --- | --- | --- |
| EfficientNet-B5 | Others | 0.58 | 0.69 | 0.63 | 0.62 |
|  | ABMR | 0.62 | 0.54 | 0.58 |  |
|  | TCMR | 0.68 | 0.61 | 0.64 |  |
| Inception V3 | Others | 0.63 | 0.64 | 0.64 | 0.62 |
|  | ABMR | 0.60 | 0.66 | 0.63 |  |
|  | TCMR | 0.64 | 0.56 | 0.59 |  |
| ResNet50 | Others | 0.59 | 0.61 | 0.60 | 0.62 |
|  | ABMR | 0.61 | 0.62 | 0.62 |  |
|  | TCMR | 0.67 | 0.63 | 0.65 |  |
| Ensemble  (at patch level) | Others | 0.61 | 0.66 | 0.63 | 0.64 |
|  | ABMR | 0.64 | 0.63 | 0.63 |  |
|  | TCMR | 0.70 | 0.62 | 0.66 |  |
| Ensemble  (at patient level) | Others | 0.80 | 0.74 | 0.77 | 0.70 |
|  | ABMR | 0.43 | 0.67 | 0.52 |  |
|  | TCMR | 0.69 | 0.61 | 0.65 |  |

Supplement Table 5. Distribution of the patient characteristics in the graft loss model.

| Characteristics | Graft loss(N=37) | Graft survival(N=54) |
| --- | --- | --- |
| Male sex | 22(59) | 33(61) |
| Age | 38(34,47) | 39.5(32.8,48.5) |
| Months from transplantation to biopsy | 8.7(5.3,33) | 6.4(2.3,15) |
| Months of follow-up after biopsy | 0.6(0,2.8) | 31.4(17.7,51) |
| Treatment response | 2(5) | 37(69) |
| Rejection subtyping |  |  |
| TCMR | 19(51) | 35(65) |
| ABMR | 8(22) | 16(30) |
| Mixed rejection | 10(27) | 3(6) |
| DSA |  |  |
| Absent | 26(70) | 38(70) |
| HLA I class | 0 | 1(2) |
| HLA II class | 3(8) | 3(6) |
| HLA I&II class | 1(3) | 2(4) |
| No available | 7(19) | 10(19) |
| C4d score |  |  |
| 0 | 32(86) | 44(81) |
| 1 | 3(8) | 3(6) |
| 2 | 0 | 3(6) |
| 3 | 2(5) | 4(7) |

*TCMR, T cell-mediated rejection; ABMR, antibody-mediated rejection; DSA, donor-specific antibody.*

Supplement Table 6. Distribution of the patient characteristics in the treatment response model.

| Characteristics | Response (N=57) | No response (N=68) |
| --- | --- | --- |
| Male sex | 35(61) | 47(69) |
| Age | 40(33.5,50) | 39.5(33,52) |
| Months from transplantation to biopsy | 10(2.6,33,1) | 13.9(5.5,47) |
| Months of follow-up after biopsy | 19.1(5,37.5) | 3.2(0.5,12.8) |
| Graft loss | 7(12) | 41(60) |
| Rejection subtyping |  |  |
| TCMR | 28(49) | 29(43) |
| ABMR | 25(44) | 21(31) |
| Mixed rejection | 4(7) | 18(26) |
| DSA |  |  |
| Absent | 43(75) | 46(68) |
| HLA I class | 0 | 1(1) |
| HLA II class | 4(7) | 6(9) |
| HLA I&II class | 2(4) | 1(1) |
| No available | 8(14) | 14(21) |
| C4d score |  |  |
| 0 | 37(65) | 47(69) |
| 1 | 10(18) | 9(13) |
| 2 | 6(11) | 5(7) |
| 3 | 4(7) | 7(10) |

*TCMR, T cell-mediated rejection; ABMR, antibody-mediated rejection; DSA, donor-specific antibody.*
